# Ameliorative inhibition of sirtuin 6 by imidazole derivative triggers oxidative stress-mediated apoptosis associated with Nrf2/Keap1 signaling in non-small cell lung cancer cell lines

**DOI:** 10.3389/fphar.2023.1335305

**Published:** 2024-01-03

**Authors:** Uma Maheswara Rao Dindi, Sameer Al-Ghamdi, Naif Abdurhman Alrudian, Salman Bin Dayel, Abdulwahab Ali Abuderman, Mohammed Saad Alqahtani, Nasraddin Othman Bahakim, Thiyagarajan Ramesh, Ravikumar Vilwanathan

**Affiliations:** ^1^ Cancer Biology Laboratory, Department of Biochemistry, School of Life Sciences, Bharathidasan University, Tiruchirappalli, Tamil Nadu, India; ^2^ Department of Family and Community Medicine, College of Medicine, Prince Sattam Bin Abdulaziz University, Al-Kharj, Saudi Arabia; ^3^ Dermatology Unit, Internal Medicine Department, College of Medicine, Prince Sattam Bin Abdulaziz University, Al-Kharj, Saudi Arabia; ^4^ Department of Basic Medical Sciences, College of Medicine, Prince Sattam Bin Abdulaziz University, Al-Kharj, Saudi Arabia; ^5^ Department of Internal Medicine, College of Medicine, Prince Sattam Bin Abdulaziz University, Al-Kharj, Saudi Arabia

**Keywords:** Epigenetics, SIRT6, HDAC, HDACi, KEAP1, Nrf2

## Abstract

**Background:** Redox homeostasis is the vital regulatory system with respect to antioxidative response and detoxification. The imbalance of redox homeostasis causes oxidative stress. Nuclear factor-erythroid 2 p45-related factor 2 (Nrf2, also called Nfe2l2)/Kelchlike ECH-associated protein 1 (Keap1) signaling is the major regulator of redox homeostasis. Nrf2/Keap1 signaling is reported to be involved in cancer cell growth and survival. A high level of Nrf2 in cancers is associated with poor prognosis, resistance to therapeutics, and rapid proliferation, framing Nrf2 as an interesting target in cancer biology. Sirtuins (SIRT1-7) are class III histone deacetylases with NAD + dependent deacetylase activity that have a remarkable impact on antioxidant and redox signaling (ARS) linked with Nrf2 deacetylation thereby increasing its transcription by epigenetic modifications which has been identified as a crucial event in cancer progression under the influence of oxidative stress in various transformed cells. SIRT6 plays an important role in the cytoprotective effect of multiple diseases, including cancer. This study aimed to inhibit SIRT6 using an imidazole derivative, Ethyl 2-[5-(4-chlorophenyl)-2-methyl-1-H-Imidazole-4-yl] acetate, to assess its impact on Nrf2/Keap1 signaling in A549 and NCI-H460 cell lines.

**Method:** Half maximal inhibitory concentration (IC_50_) of Ethyl 2-[5-(4-chlorophenyl)-2-methyl-1-H-Imidazole-4-yl] acetate was fixed by cell viability assay. The changes in the gene expression of important regulators involved in this study were examined using quantitative real-time PCR (qRT-PCR) and protein expression changes were confirmed by Western blotting. The changes in the antioxidant molecules are determined by biochemical assays. Further, morphological studies were performed to observe the generation of reactive oxygen species, mitochondrial damage, and apoptosis.

**Results:** We inhibited SIRT6 using Ethyl 2-[5-(4-chlorophenyl)-2-methyl-1-H-Imidazole-4-yl] acetate and demonstrated that SIRT6 inhibition impacts the modulation of antioxidant and redox signaling. The level of antioxidant enzymes and percentage of reactive oxygen species scavenging activity were depleted. The morphological studies showed ROS generation, mitochondrial damage, nuclear damage, and apoptosis. The molecular examination of apoptotic factors confirmed apoptotic cell death. Further, molecular studies confirmed the changes in Nrf2 and Keap1 expression during SIRT6 inhibition.

**Conclusion:** The overall study suggests that SIRT6 inhibition by imidazole derivative disrupts Nrf2/Keap1 signaling leading to oxidative stress and apoptosis induction.

## 1 Introduction

Lung cancer is the leading cause of cancer deaths worldwide. Lung cancer is classified into two main types, non-small cell lung carcinoma (NSCLC, 85%) and small-cell lung carcinoma (SCLC, 25%). NSCLC is the most common with three main types including adenocarcinoma (40%), squamous cell carcinoma (25%–30%), and large cell carcinoma (5%–10%)(Alduais et al., 2023). Various phenotypic manifestations are associated with the onset of cancer, while oxidative stress plays a crucial role in the progression of tumorigenesis by impairing important pathways in DNA repair, cell survival, and proliferation, favoring genetic and/or epigenetic dysregulation of oncogenes and tumor suppressor genes (Tossetta and Marzioni, 2023). In normal cells, controlled production of reactive oxidants is delivered to fulfill the much-needed purpose of regulating signaling pathways which are counterbalanced by an antioxidant defense system, ensuring adequate response whenever needed by the body (Ma, 2013). Oxidative stress is generated due to an imbalance between highly reactive molecules called reactive oxygen species (ROS), such as superoxide (O2−), hydroxyl radical (OH−), and hydrogen peroxide (H_2_O_2_), and antioxidants, namely, superoxide dismutase, catalases, thioredoxins, peroxiredoxins, reductases, and peroxidases (Tossetta et al., 2023). ROS exposure prompts the production of antioxidant enzymes that neutralize ROS. ROS can also be neutralized by non-enzymatic compounds such as glutathione, coenzyme Q, and lipoic acid which prevents oxidative stress by quenching ROS (Fuchs-Tarlovsky, 2013). Oxidative stress thus plays a pivotal role in determining cell fate to promote normal cell death in response to excessive ROS production. However, in carcinogenesis, a high amount of ROS promotes defective signaling factors through the deregulation of biomolecules. Therefore, the factors that could regulate cell survival pathways in transformed cells in response to oxidative stress stimuli are actively considered as potential prognostic factors and possible therapeutic targets.

Nuclear factor erythroid-2-related factor 2/Kelch-like ECH-associated protein 1 (Nrf2/Keap1) is the major regulator of cellular homeostasis in response to xenobiotic and oxidative stress (Wu et al., 2019). Nrf2 initiates cellular rescue pathways against oxidative injury, inflammation/immunity, apoptosis, and carcinogenesis. Under normal conditions, Nrf2 is constitutively expressed and degraded in the cytoplasm. Keap1 mediates the polyubiquitination and degradation of Nrf2 through the Cul3 E3 ligase complex (Schmidt et al., 2016). In oxidative stress, Keap1 cysteine residues undergo a conformational change, which disassociates Keap1 binding to Nrf2, and this prevents degradation of Nrf2, leading to the accumulation of Nrf2 in the cytosol (Kobayashi et al., 2006). Nrf2 moves to the nucleus and binds to antioxidant response elements (AREs) in the promoter region of antioxidant regulatory genes and protects cells from oxidative damage by transcriptionally activating antioxidant genes (Leung et al., 2019). Nrf2 protects both normal cells and cancer cells from cellular stress (Yoo et al., 2012). In normal cells, Nrf2 activation prevents DNA damage and mutations which can initiate carcinogenesis. At the same time, Nrf2 in transformed cells supports tumor progression by protecting against oxidative damage (DeBlasi and DeNicola, 2020). The constitutive expression of Nrf2 creates a redox environment that facilitates tumor growth and promotes resistance to chemotherapy. High levels of Nrf2 are associated with poor prognosis (Zhang, 2010; Lu et al., 2017). Dysregulation of Nrf2/Keap1 is reported in several tumors, including NSCLC. The functional loss of Keap1 leads to the constitutive expression of Nrf2 in about 19% of tumors and up to 50% of NSCLC cell lines (Leone et al., 2015). Nrf2 upregulation and Keap1 downregulation are common abnormalities in NSCLC and are associated with poor prognosis (Solis et al., 2010).

Controlled Nrf2 signaling is regulated at the transcriptional, translational, and post-translational levels. Earlier genetic changes have been reported to be involved in the regulation of Nrf2/Keap1, but in recent times epigenetic alterations have been found to control the Nrf2 signaling pathway (Bhattacharjee and Dashwood, 2020). Studies of epigenetic modification by inhibiting histone deacetylases (HDACs) found Nrf2-controlled expression at the post-transcriptional level (Dong et al., 2017). HDACs are a group of histone-modifying enzymes, which function by removal of the acetyl group from ε-N–acetyl lysine amino acid residues from histones. In humans, a total of 18 HDACs are reported and these are classified into the classical and sirtuin families. These two families are further categorized into four classes. Sirtuins are class III histone deacetylases with highly conserved NAD + domain. Sirtuins are known to have similarities with yeast *Saccharomyces cerevisiae* protein Sir2 (silent information regulator 2). In humans, there are seven distinct types of sirtuins, each with variations in cellular localization, enzymatic activities, and target proteins. SIRT1 and SIRT6 are in the nucleus, while SIRT2 is cytosolic. Whereas SIRT3, SIRT4, and SIRT5 are found in mitochondria (Khan et al., 2021).

Among the sirtuins, SIRT6 has a dual role in tumorigenesis and tumor suppression. This depends on the specific context and the type of cancer (Fiorentino et al., 2021). We previously reported that the knockdown of SIRT6 induced cell cycle arrest and apoptosis in NSCLC cell lines (Krishnamoorthy and Vilwanathan, 2020). Oxidative stress is reported to be involved in tumor initiation and progression (Tossetta and Marzioni, 2022). SIRT6 has been reported to play a prominent role in promoting cancer cell survival under oxidative stress, confirming its role in redox-related cellular homeostasis. SIRT6 acts as a key regulator of oxidative stress and counteracts oxidative stress by regulating the expression and activity of antioxidant enzymes, such as superoxide dismutase (SOD), catalase (CAT), and ROS detoxification. This provides a survival advantage to cancer cells exposed to high ROS levels (Liu et al., 2023). The Nrf2/Keap1 pathway is a pivotal player in the signaling cascade responsible for the resistance of oxidative damage. Recent evidence suggests that Nrf2 has an aberrant activation associated with poor prognosis, can inhibit apoptosis, and promotes tumor proliferation (Wu et al., 2019). Considering the key regulating role of SIRT6 on oxidative stress in transformed cells, SIRT6 might be a potential target for countering Nrf2/Keap-mediated cellular rescue pathways in tumor cells. Generally, heterocyclic compounds with nitrogen atoms have a broad range of pharmaceutical activities (Ali et al., 2017). According to reports, 59% of small-molecule agents approved by the FDA have nitrogen-containing heterocyclic rings (Sharma et al., 2021). Imidazole is an aromatic compound with five-membered heterocyclic rings and two nitrogen atoms that preserve an important class of therapeutic agents in current medicinal science (Guda et al., 2017). Imidazole’s pharmacological properties, in particular anticancer activity, attracted the development of imidazole derivative-based anticancer drugs with increasing efficiency and lowering of the side effects (Alghamdi et al., 2021). The modifications by the addition of substituents to the imidazole ring can alter its pharmacokinetic properties, allowing for improved interactions of the imidazole derivative. Recently, the pyrrole-pyridinimidazole derivative has been reported as a potent SIRT6 inhibitor with a promising non-competitive inhibition profile in pancreatic cancer cells (Song et al., 2023). We have also performed a virtual screening study on binding pattern analysis of a series of imidazole derivatives for their inhibitory effect on sirtuins (Dindi et al., 2023). Considering SIRT6 inhibition regulates Nrf2 expression in transformed cells, herein, we evaluate the effect of SIRT6 inhibition using Ethyl 2-[5-(4-chlorophenyl)-2-methyl-1-H-Imidazole-4-yl) acetate on the Nrf2/Keap1 signaling pathway in NSCLC cell lines.

## 2 Materials and methods

### 2.1 Reagents and antibodies

Cell culture reagents Dulbecco’s Modified Eagle’s Medium (DMEM) (AL151A), fetal bovine serum (FBS) (RM10432), 10 × Phosphate Buffered Saline (PBS, pH 7.4) (TL1099-500 ML), 10 × Trypsin-EDTA (TCL144-100 ML), 10 × antibiotic antimycotic solution (penicillin, streptomycin, and amphotericin B) (A002-100 ML), and Dimethyl Sulphoxide (DMSO) (cell-culture tested) (TC185-100 ML) were purchased from Hi-Media Laboratories, Mumbai, India. RNAiso Plus (Total RNA extraction reagent) (9108). A Prime Script RT reagent kit (Perfect Real Time) (RR037A) and TB Green Premix Ex Taq II (Tli RNase H Plus) (RR820A) were purchased from Takara Bio Inc. (Japan). A radioimmunoprecipitation assay (RIPA) lysis buffer system (sc-24948) for protein isolation was purchased from Santa Cruz (CA, United States). Nitrocellulose membrane (0.45µm, cat. log1620115) and Precision Plus Protein™ Kaleidoscope™ Prestained Protein Standards (#1610375) were purchased from Bio-Rad Laboratories (Hercules, California, United States). PageRuler Prestained Protein Ladder (cat.log 26616) was purchased from Thermo Fisher Scientific (Waltham, MA, United States). Antibodies against SIRT6 (D8D12) and cleaved caspase-3 (Asp175) (5A1E) were purchased from Cell Signaling Technology (Danvers, MA, United States). Caspase-3 antibody (sc-7272), caspase-9 antibody (sc-70505), and cytochrome-c (sc-13156) were purchased from Santa Cruz (CA, United States). β-actin (MAB8929-SP) was purchased from Novus Biologicals (Briarwood, CO, United States). Nrf2 polyclonal antibody (BT-AP06174) was purchased from Bioassay Technology Laboratory. Keap1 polyclonal antibody (ABP52949) was purchased from Abbkine (Georgia, United States). The secondary antibodies, such as goat anti-mouse IgG H&L (Alkaline Phosphatase) (ab97020) and goat anti-rabbit IgG H&L (Alkaline Phosphatase) (ab6722), were purchased from Abcam (Cambridge, United Kingdom) and 5-bromo-4-chloro-3-indolyl phosphate (BCIP)/nitro blue tetrazolium (NBT) substrate (B1911-100 ML) was purchased from Sigma-Aldrich Co. (United States).

### 2.2 Cell culture

The human non-small cell lung cancer cell lines A549 (Adenocarcinoma) and NCI-H460 (large cell carcinoma) were purchased from the National Centre for Cell Science (NCCS), Pune, India. The cell lines were cultured using a complete medium which includes, DMEM, 10% FBS, and 1% antibiotic antimycotic solution (penicillin, streptomycin, and amphotericin B). The cultured cell lines were incubated in a CO_2_ incubator at an atmospheric temperature of 37°C and supplied with 5% CO_2_.

### 2.3 3-(4,5-Dimethylthiazol-2-yl)-2,5-diphenyltetrazolium bromide (MTT) assay

Half maximal inhibitory concentration (IC_50_) is a quantitative measure of the amount of a substance (e.g., drug) needed to inhibit biological processes or biological components by 50%. An MTT assay is a well-known assay to determine the IC_50_. The stock solution of an imidazole derivative was prepared in cell culture grade DMSO. The working solution of an imidazole derivative was prepared in an incomplete medium and this was used to determine the IC_50_. The cell lines grown in monolayer were trypsinized and pelleted. The cell pellet was suspended in a complete medium. The cells were then counted using the trypan blue and the hemacytometer. Approximately 1 × 10^4^ cells were seeded in 96-well plates and incubated overnight in a CO_2_ incubator. After incubation, the old media was aspirated, and incomplete media was added to the wells. The cells were then treated with a working solution of Ethyl 2-[5-(4-chlorophenyl)-2-methyl-1-H-Imidazole-4-yl) acetate with a dosage ranging from 50 μM to 500 µM and incubated for 24 h. After the treatment period, 20 µL of 5 mg/mL concentration of MTT was added to the wells and incubated for 4 h. Later, the media was removed from the wells and 200 μL of dimethyl sulphoxide (DMSO) was added to dissolve the formazan crystals. After 30 min of incubation, the intensity of the purple color formed was measured by an ELISA plate reader (Bio-Rad Laboratories, Hercules, CA, United States) at 595 nm. The percentage of viable cells was calculated by using the following formula:

% Cell Viability=Mean OD of treated cells/ Mean OD of control cells×100

### 2.4 Quantitative real-time PCR (qRT-PCR)

The A549 and NCI-H460 cells were seeded into Petri plates and grown in a complete medium. Once the growth reached 80% confluency, the cells proceeded for treatment. The IC_50_ of Ethyl 2-[5-(4-chlorophenyl)-2-methyl-1-H-Imidazole-4-yl) acetate in incomplete media was used for treatment. The control cells were maintained in an incomplete medium without Ethyl 2-[5-(4-chlorophenyl)-2-methyl-1-H-Imidazole-4-yl) acetate. After the treatment period, total RNA was isolated from untreated (control) and treated A549 and NCI-H460 cell lines. The quantity and purity of RNA are determined using a BioPhotometer (Eppendorf, Hamburg, Germany) and 1 μg of total RNA was taken for cDNA construction using PrimeScript RT reagent kit as per the manufacturer’s instructions. The qRT-PCR for gene expression studies was performed in the StepOnePlus real-time PCR system (Applied Biosystems, Thermo Fisher Scientific, MA, United States) using 2 × SYBR green master mix (TB Green Premix Ex Taq II (Tli RNase H Plus). The sample preparation was done according to the instructions in the datasheet provided by the manufacturer and 25 ng of cDNA was used for each 10 μL reaction. The PCR condition includes initial denaturation at 94°C for 5 min, followed by 40 cycles with denaturation at 94°C for 30s, annealing at 54.9°C–60°C for 30 s (depending on specific gene), and melt curve stage conditions: 95°C for 15s, 60°C for 60 s, and 95°C for 15 s. The relative mRNA expression in fold change was calculated using the 2^(-ΔΔCt) method. Glyceraldehyde 3-phosphate dehydrogenase (GAPDH) was used as an endogenous control. The list of primers used in this study is provided in Table 1.

**TABLE 1:** List of primers used in the studies.

S. No	Genes	Primer sequence 5′–3′	Annealing.Temp (^o^C)	Product Size (bp)
1	GAPDH	F**′** P: ATG​GGG​AAG​GTG​AAG​GTC​G	60	107
		R**′** P: GGT​CAT​TGA​TGG​CAA​CAA​TAT​C		
2	Keap1	F**′** P: TAC​TTC​CGA​CAG​TCG​CTC​AG	57.5	194
		R**′** P: GGG​TTG​TAA​CAG​TCC​AGG​GC		
3	Nrf2	F**′** P: CAA​GTC​CCA​GTG​TGG​CAT​CA	57.5	188
		R**′** P: CCC​CTG​AGA​TGG​TGA​CAA​GG		
4	Sirtuin 6	F**′** P: TCCCATTGTCTAGCCTCA	58.1	181
		R**′** P: GATGTCGGTGAATTACGC		
5	Caspase 3	F**′** P: GCA​AGT​TAC​AGT​GAT​GCT​GTG​C	54.9	166
		R**′** P: CCA​TGC​CCA​CAG​ATG​CCT​AA		
6	Caspase 9	F**′** P: CAT​CCC​AGG​AAG​GCA​ACA​AG	54.9	131
		R**′** P: GGG​AAG​CAT​GGC​TAG​GAC​TC		

### 2.5 Western blot analysis

The cell lines grown in monolayer were treated with Ethyl 2-[5-(4-chlorophenyl)-2-methyl-1-H-Imidazole-4-yl) acetate in incomplete media and untreated cell lines maintained in incomplete media were used as control samples. After the treatment period, the cells were washed with ice-cold PBS twice and lysed with a Radioimmunoprecipitation assay (RIPA) lysis buffer system supplemented with protease and phosphatase inhibitor cocktails. The cell lysate was centrifuged at 12,000 g for 20 min at 4°C and a supernatant containing the whole protein was collected. The protein samples were quantified by Lowry’s method and 50 μg of protein samples were used for Western blot analysis. Protein was separated by sodium dodecyl-sulfate polyacrylamide gel electrophoresis (SDS-PAGE) (10% and 12% gels), and separated proteins were transferred onto the nitrocellulose membrane by wet transfer. The membrane was blocked with 5% skimmed milk for 2 h. After the incubation, the membrane was washed with 1 × TBST for 5 min three times. After the TBST wash, the membrane was incubated overnight at 4°C with specific primary antibodies dilution (1:1000) prepared in 5% BSA in TBST. After overnight incubation, the membrane was washed three times with 1 × TBST for 5 min each. The alkaline phosphatase-conjugated secondary antibody (1:5000) specific to the primary antibody prepared in 5% BSA in TBST was added and incubated for 4 h at 4°C. Then the membrane was washed three times with 1 × TBST for 5 min each. The blots were developed with BCIP/NBT chromogenic substrate, and the developed blots were scanned using a Canon imageCLASS MF244dw. ImageJ software (National Institutes of Health, Bethesda, MD, United States) was used to measure the intensity of the bands. The protein expression in fold change was calculated after normalizing with the endogenous control beta-actin. The complete details of the antibodies used in this study are provided in Table 2.

**TABLE 2:** List of antibodies used in the studies.

S. No	Antibody	Molecular weight (kDa)	Brand	Cat. Log
1	β-Actin	45	Novus Biologicals	MAB8929-SP
2	Nrf2 Polyclonal Antibody	72–100	BT LAB	BT-AP06174
3	Keap1 Polyclonal Antibody	70	Abbkine	ABP52949
4	SirT6 (D8D12) Rabbit mAb	42, 36	Cell Signaling Technology	#12486
5	Cleaved Caspase-3 (Asp175) (5A1E)	17, 19	Cell Signaling Technology	#9664
6	caspase-3 Antibody (E−8)	32	Santa Cruz Biotechnology	sc-7272
7	caspase-9 Antibody (3C122)	46, 35	Santa Cruz Biotechnology	sc-70505
8	cytochrome c (A-8)	15	Santa Cruz Biotechnology	sc-13156
9	Goat Anti-Mouse IgG H&L		Abcam	ab97020
10	Goat Anti-Rabbit IgG H&L		Abcam	ab6722

### 2.6 Acridine orange/ethidium bromide (AO/EtBr) dual staining

To visualize apoptotic morphological changes by acridine orange/ethidium bromide dual staining, approximately 1 × 10^5^ cells were seeded in a 6-well plate. After reaching 60% confluency, the cells were treated with an IC_50_ of Ethyl 2-[5-(4-chlorophenyl)-2-methyl-1-H-Imidazole-4-yl) acetate. After 24 h of incubation, the cells were stained with 50 μL/mL of AO/EtBr and incubated at 37°C with 5% CO_2_ for 20 min. After the incubation, the cells were washed with 1 × PBS to remove the extra dye, and the morphological changes were visualized using the 20 × magnification under a fluorescence microscope (Floid cell imaging station).

### 2.7 Detection of cell death using propidium iodide staining

Approximately 1 × 10^5^ cells were counted and seeded in a 6-well plate. When the cell growth reached 60%, the cells were treated for a treatment period of 24 h with an IC_50_ of Ethyl 2-[5-(4-chlorophenyl)-2-methyl-1-H-Imidazole-4-yl) acetate and incubated at 37°C with 5% CO_2._ After the treatment period, each well was stained with 50 μL/mL of propidium iodide and incubated for 20 min. The morphological changes were visualized and photographed using 20 × magnification under a fluorescence microscope (Floid cell imaging station).

### 2.8 Reactive oxygen species (ROS) detection by DCFH-DA

The ROS generation in A549 and NCI-H460 cells was observed by fluorescence microscopy. For morphological assessment of ROS generation, approximately 1 × 10^5^ cells were seeded in a 6-well plate. After reaching 60% confluency, the cells were treated with an IC_50_ of Ethyl 2-[5-(4-chlorophenyl)-2-methyl-1-H-Imidazole-4-yl) acetate and incubated for a treatment period of 24 h. After the treatment period, the cells were stained with 40 μM DCFH-DA and incubated at 37°C with 5% CO_2_ for 20 min. After the incubation, 1 × PBS was used to remove the extra dye, and morphological changes were visualized and captured using the 20 × magnification under a fluorescence microscope (Floid cell imaging station).

### 2.9 Mitochondrial membrane potential (ΔΨm) determination by Rhodamine-123

The changes in mitochondrial membrane potential (ΔΨm) in A549 and NCI-H460 cell lines were assessed using rhodamine-123 dye. Approximately, 1 × 10^5^ cells were seeded in each well of a 6-well plate. After reaching 60% confluency, the cells were treated with an IC_50_ of Ethyl 2-[5-(4-chlorophenyl)-2-methyl-1-H-Imidazole-4-yl) acetate for a treatment period of 24 h. After the treatment period, each well was stained with 50 μL of rhodamine-123 dye (10 mg/mL) and incubated at 37°C with 5% CO_2_ for 20 min. After 20 min incubation, the stained cells were washed with 1 × PBS to remove the excess dye. The fluorescence images of the morphological changes were captured using the 20 × magnification under a fluorescence microscope (Floid cell imaging station).

### 2.10 Determination of nuclear fate by Hoechst staining

To evaluate whether Ethyl 2-[5-(4-chlorophenyl)-2-methyl-1-H-Imidazole-4-yl) acetate induced apoptosis in A549 and NCI-H460 cell lines, a Hoechst 33258 staining was performed to observe the nuclear morphological changes which occurred during the apoptosis. Approximately 1 × 10^5^ cells were counted and seeded in a 6-well plate. Once the cell growth reached 60%, the cells were treated with an IC_50_ of Ethyl 2-[5-(4-chlorophenyl)-2-methyl-1-H-Imidazole-4-yl) acetate for a treatment period of 24 h and incubated at 37°C with 5% CO_2._ Later, the cells were stained with 50 μL/mL of Hoechst 33258 and incubated for 20 min. The excess staining was then removed by washing with 1 × PBS. The changes in the nuclei of cells were visualized and photographed using the 20 × magnification under a fluorescence microscope (Floid cell imaging station).

### 2.11 Assessment of the antioxidant and radical scavenging activity

Biochemical analysis of enzymatic antioxidants such as catalases (CAT) and glutathione peroxidases (GPx), non-enzymatic antioxidant glutathione (GSH), and percentage radical scavenging activity (%RSA) was performed with cell homogenate of control (untreated) and Ethyl 2-[5-(4-chlorophenyl)-2-methyl-1-H-Imidazole-4-yl) acetate treated A549 and NCI-H460 cell lines. The cells grown in monolayer were treated with an IC_50_ of Ethyl 2-[5-(4-chlorophenyl)-2-methyl-1-H-Imidazole-4-yl) acetate, and untreated cells were used as control. After a treatment period of 24h, the cells were lysed with RIPA lysis buffer and then centrifuged at 12,000 rpm for 20 min at 4°C. The supernatant is collected and used for antioxidant studies.

To determine the GSH levels, the cell lysate was deproteinated by adding 10% trichloroacetic acid (TCA). Later, the contents were centrifuged at 12,000 g for 10 min in a 4°C cooling centrifuge and the supernatant was collected. Furthermore, 1 mL of supernatant was added to 4 mL of 0.3 M phosphate solution and 0.5 mL of 2 mg/5 mL concentration of 5,5′-dithio-bis-(2-nitrobenzoic acid) (DTNB). DTNB reacts with GSH to form a yellow-colored chromophore, 5-nitro-2-thiobenzoic acid (TNB), with absorbance maxima at 412 nm. After 5 min of incubation at room temperature, the absorbance at 412 nm was recorded using a UV-visible spectrophotometer. Except for the cell homogenate, all the other reagents in the reaction were used as blank.

The GPx activity assay was performed using the Paglia and Valentine (1967) method (Paglia and Valentine, 1967). The reaction mixture contained 0.4 mL of 0.4 M phosphate buffer (pH 7), 0.1 mL sodium azide (16 mM), 0.2 mL reduced glutathione (4 mM), 0.1 mL of H_2_O_2_ (2.5 mM), and 0.2 mL of homogenate. The mixture was incubated at room temperature for 0s, 90s, and 180s. The reaction was arrested by adding 0.5 mL of 10% TCA, and the tubes were centrifuged at 2,000 rpm for 5min. To 1 mL of the supernatant, 3 mL of phosphate disodium hydrogen phosphate (0.3 mM) and 1 mL of DTNB (0.04%) were added. The color developed was read in a UV-visible spectrophotometer at 412 nm. The reaction mixer except the cell homogenate was considered blank.

Catalase activity was determined by the rate of decomposition of H_2_O_2_ (Aebi, 1984). To 0.1 mL of homogenate, 1 mL of phosphate buffer (50 mM, pH 7) and 0.5 mL of H_2_O (20 mM) were added and incubated for 0 min, 30 min, and 60 min. After the incubation, the reaction was stopped by adding 2 mL of potassium dichromate. The reaction mixer without a sample was taken as blank. The results were calculated from the extension coefficient of H_2_O_2_ at 240 nm in a UV-visible spectrophotometer.

A 2,2-Diphenyl-1-picrylhydrazyl (DPPH) assay was performed to determine the %RSA using the protocol from Mensor LL et al. (Mensor et al., 2001). DPPH reacts with antioxidants and is converted to a reduced form of DPPH, indicated by the change of color from deep violet (DPPH) to light yellow (DPPH). The assay was performed by adding 3 mL of ethanol, 0.5 mL of the 10% cell homogenate, and 0.3 mL of freshly prepared DPPH (0.5 mM) solution. Furthermore, 3.3 mL of ethanol and 0.5 mL of sample were used as blank, whereas 3.5 mL of ethanol and 0.3 mL DPPH were used as the control. The samples were incubated for 100 min and the change in color was read at 517 nm using a UV-visible spectrophotometer. %RSA is calculated using the following formula

$$\%\text{RSA}=100‐\left[\left(\text{Sample}\text{Abs}‐\text{Blank}\text{Abs}\right)/\text{Control}\text{Abs}\times 100\right]$$

### 2.12 Statistical analysis

The graphical presentation of the data in this study is from biological replicates and presented as the mean ± SD done using one-way ANOVA with Tukey’s multiple comparisons test by GraphPad Prism software (version 9.4.0, CA, United States). The significance is represented as *p < 0.05 and **p < 0.01, ***p < 0.001, and ****p < 0.0001.

## 3 Results

### 3.1 Cytotoxicity effect of ethyl 2-[5-(4-chlorophenyl)-2-methyl-1-H-imidazole-4-yl) acetate

**FIGURE 1:**
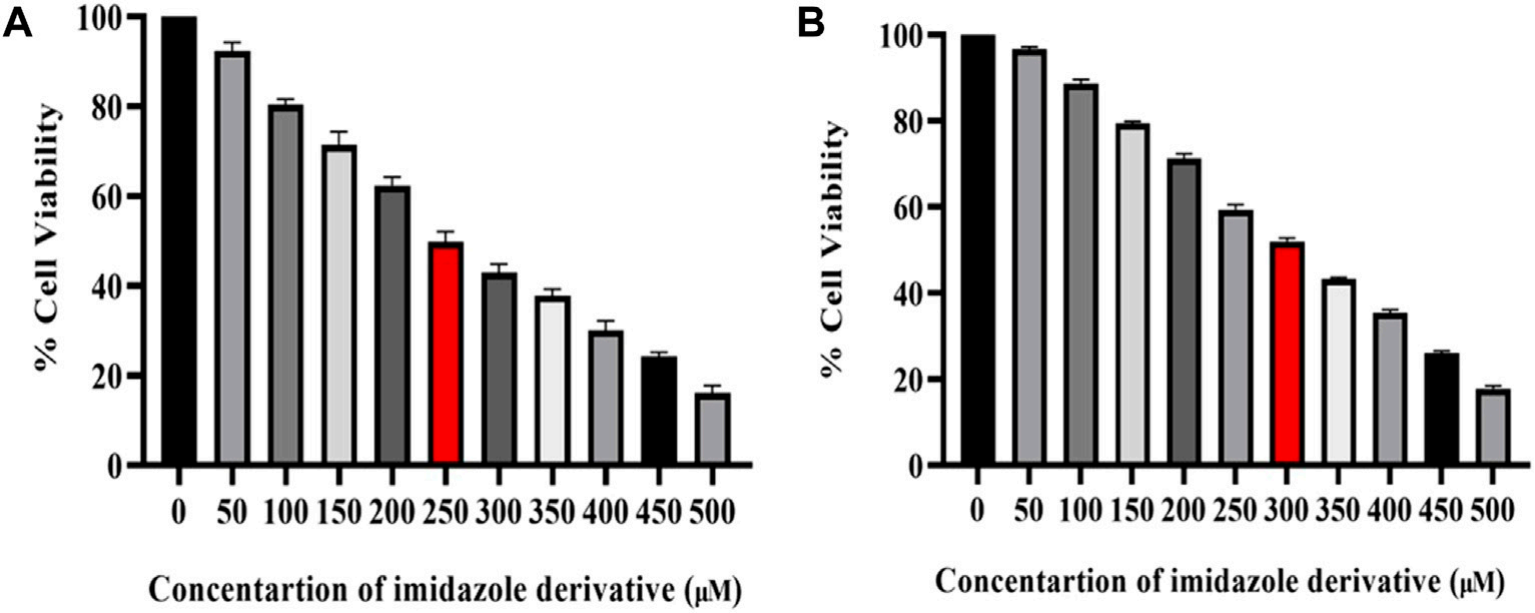
Illustration of the cytotoxicity activity of Ethyl 2-[5-(4-chlorophenyl)-2-methyl-1-H-Imidazole-4-yl] acetate in **(A)** A549 and **(B)** NCI-H460 cell lines.

The cytotoxicity of imidazole derivative Ethyl 2-[5-(4-chlorophenyl)-2-methyl-1-H-Imidazole-4-yl) acetate was determined in NSCLC cell lines A549 and NCI-H460 using an MTT assay. The cells were treated with imidazole derivative ranging from 0 μM to 500 µM concentration for 24 h. The cell viability decreased gradually with an increase in concentration. The IC_50_ was found to be 250 µM in the A549 cell line and 300 µM in the NCI-H460 cell line (Figure 1).

### 3.2 Ethyl 2-[5-(4-chlorophenyl)-2-methyl-1-H-imidazole-4-yl) acetate effect on sirtuin 6

**FIGURE 2:**
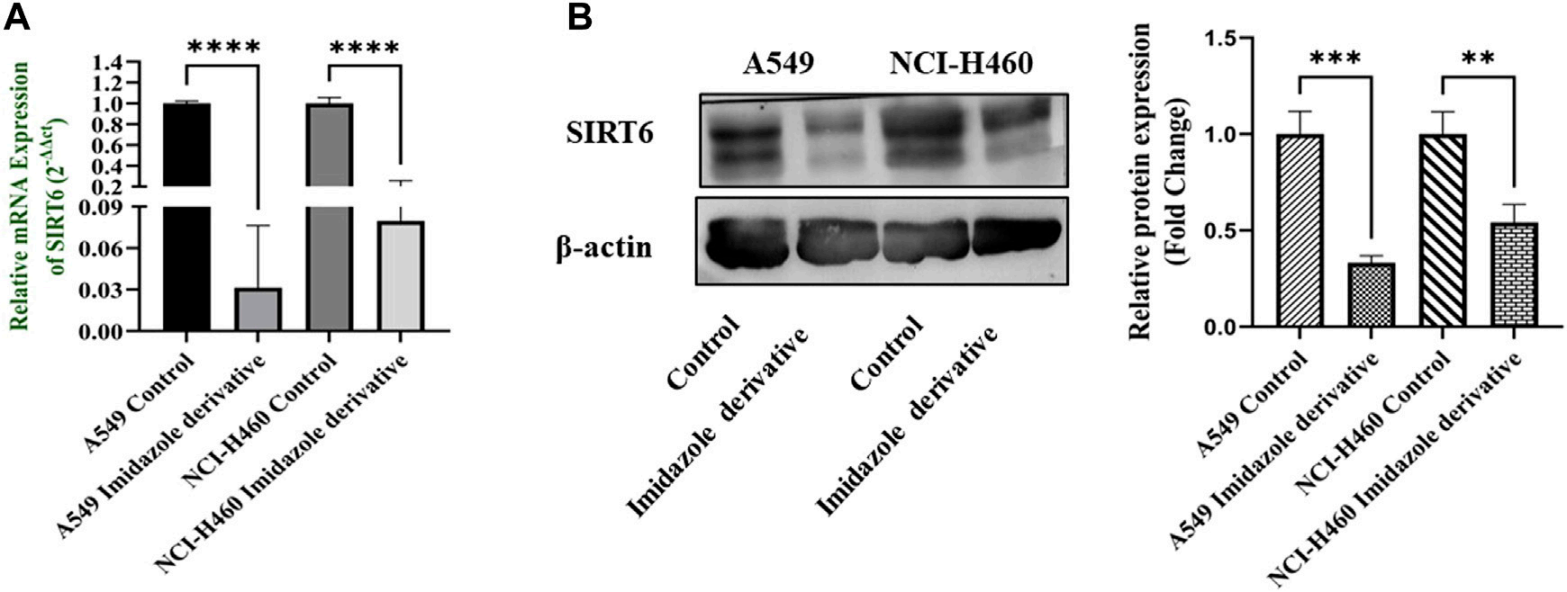
SIRT6 expression in A549 and NCI-H460 NSCLC cell lines: **(A)** Represents the qRT-PCR analysis and **(B)** Represents the Western blot analysis. GAPDH for gene expression and β-actin for protein expression were used as an internal control for normalization (Data represent mean values ±SD. **p < 0.01, ***p < 0.001 and ****p < 0.0001).

The SIRT6 expression in NSCLC cell lines, A549 and NCI-H460, was examined using Ethyl 2-[5-(4-chlorophenyl)-2-methyl-1-H-Imidazole-4-yl) acetate treated and untreated samples. The results of the gene and protein expression studies revealed that SIRT6 expression was decreased in both A549 and NCI-H460 cell lines treated with Ethyl 2-[5-(4-chlorophenyl)-2-methyl-1-H-Imidazole-4-yl) acetate compared to the control (untreated) cells (Figure 2). This indicates that Ethyl 2-[5-(4-chlorophenyl)-2-methyl-1-H-Imidazole-4-yl) acetate has an inhibitory effect on SIRT6.

### 3.3 Sirtuin 6 inhibition affects antioxidant systems

**FIGURE 3:**
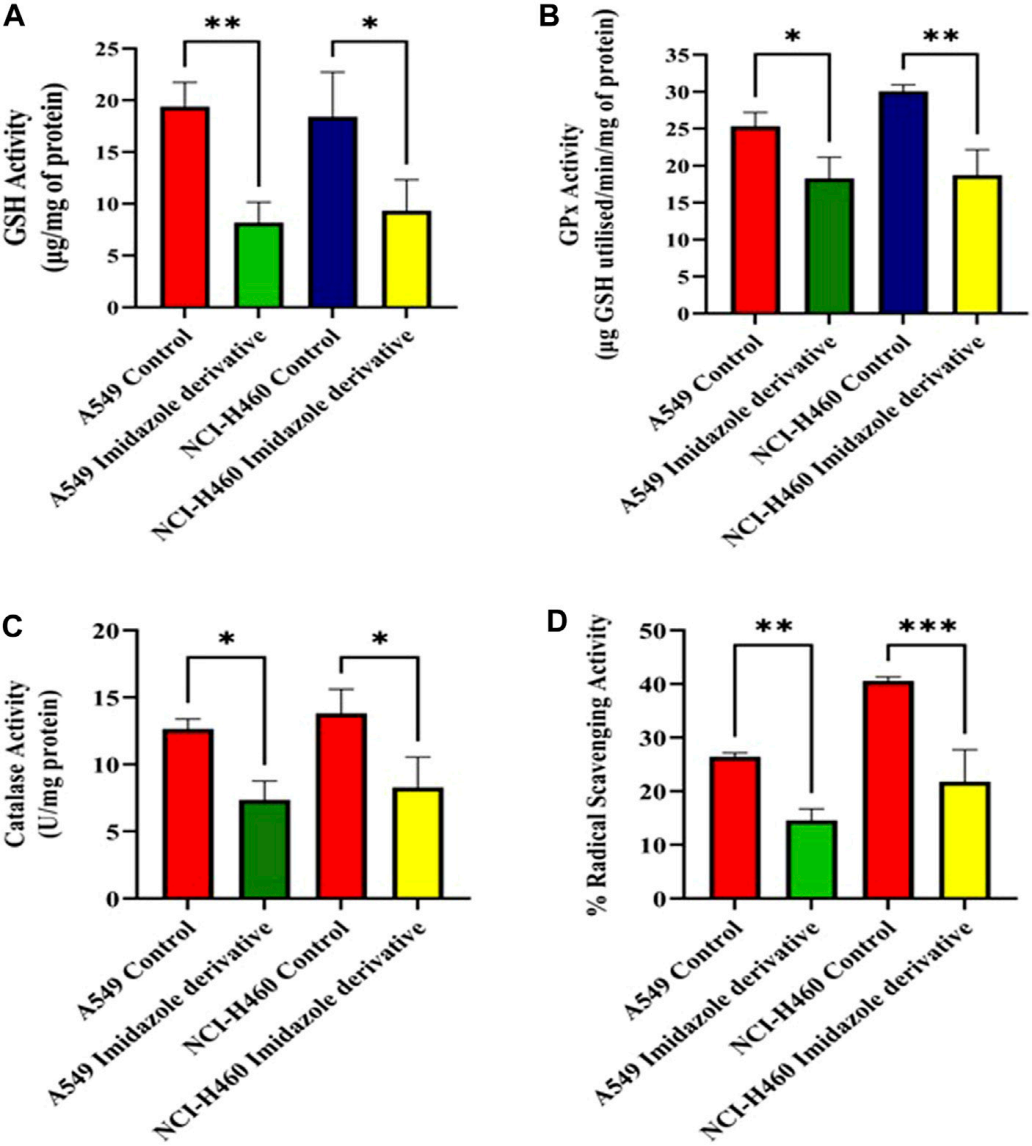
The effect of imidazole derivative Ethyl 2-[5-(4-chlorophenyl)-2-methyl-1-H-Imidazole-4-yl] acetate on antioxidant regulators **(A)** GSH Activity, **(B)** GPx Activity, **(C)** Catalase Activity, and **(D)** % Radical Scavenging Activity in A549 and NCI-H460 cell lines. (Data represent mean values ±SD. *p < 0.05, **p < 0.01, and ***p < 0.001).

We performed biochemical assays to determine the fate of antioxidant molecules upon inhibition of SIRT6 in NSCLC cell lines. We estimated the levels of both enzymatic (GPx, and CAT) and non-enzymatic antioxidant molecules (GSH). The GSH, GPx, and CAT levels were reduced in the Ethyl 2-[5-(4-chlorophenyl)-2-methyl-1-H-Imidazole-4-yl) acetate-treated cells. Further, the antioxidant activity was determined by the free radical scavenging activity using a DPPH assay. The %RSA was reduced in cells treated with the imidazole derivative compared to untreated cells (Figure 3). The antioxidant assays confirm that the SIRT6 inhibition affects the antioxidant system and free radical scavenging activity.

### 3.4 Oxidative stress impact on apoptosis

**FIGURE 4:**
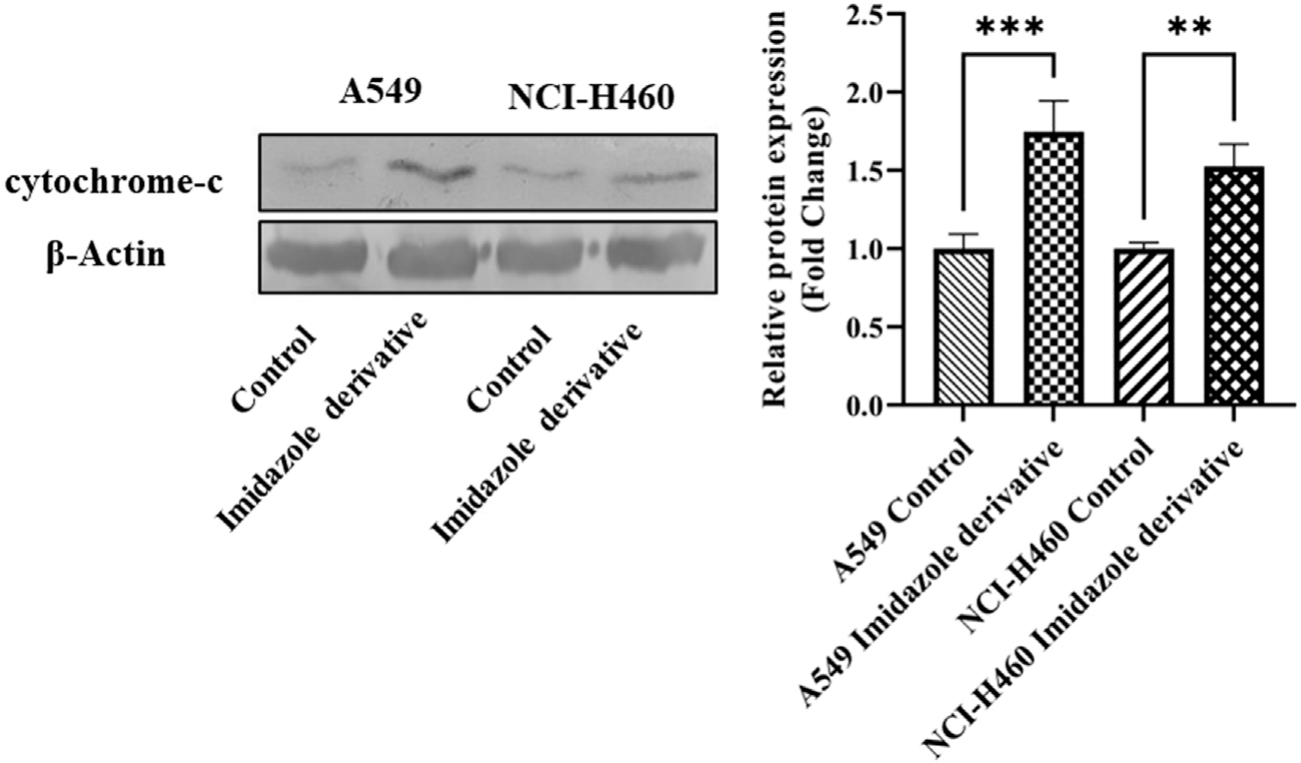
The protein expression of cytochrome-c (cyt-c) in NSCLC cell lines A549 and NCI-H460. β-actin is used as an internal control for normalization. (Data represent mean values ±SD. **p < 0.01, and ***p < 0.001).

**FIGURE 5:**
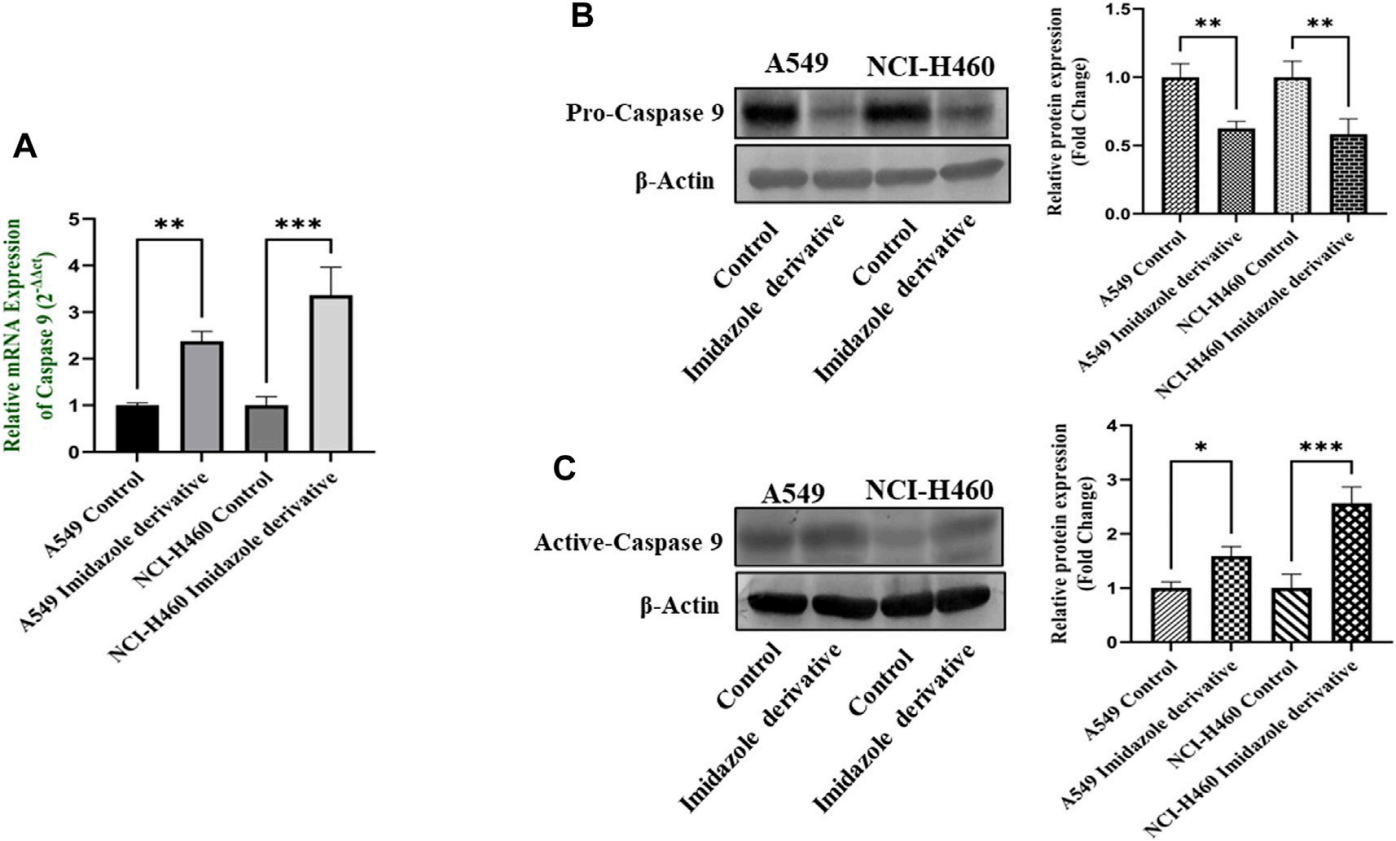
Gene and protein expression of caspase 9: **(A)** Caspase 9 gene expression, **(B)** pro-caspase 9, and **(C)** active-caspase 9 protein expression in A549 and NCI-H460 cell lines. GAPDH for gene expression and β-actin for protein expression were used as an internal control for normalization. (Data represent mean values ±SD. *p < 0.05, **p < 0.01 and ***p < 0.001).

**FIGURE 6:**
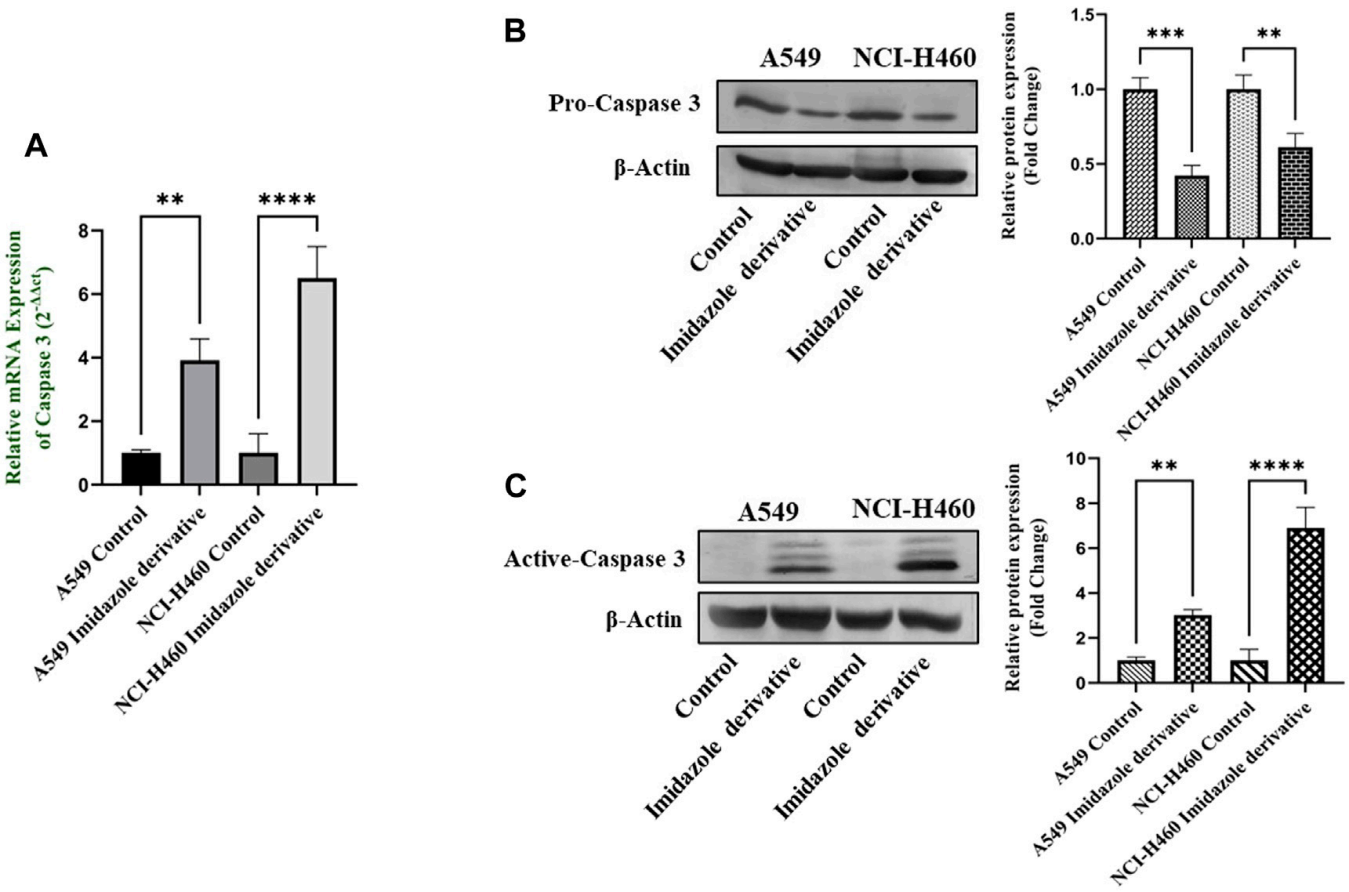
The expression of Caspase 3: **(A)** Caspase 3 gene expression, **(B)** protein expression of pro-caspase 3, and **(C)** active-caspase 3 in A549 and NCI-H460 cell lines. GAPDH for gene expression and β-actin for protein expression were used as an internal control for normalization (Data represent mean values ±SD.**p < 0.01, ***p < 0.001 and ****p < 0.0001).

The imbalance between ROS and antioxidant mechanisms causes ROS accumulation, leading to oxidative stress. The accumulation of ROS above the threshold level destroys mitochondria. As mitochondria play a significant role in the intrinsic apoptosis pathway, we investigated the downstream regulators associated with this pathway, such as cytochrome-c (cyt-c), caspase 9, and caspase 3. Elevated levels of cyt-c were observed in the Ethyl 2-[5-(4-chlorophenyl)-2-methyl-1-H-Imidazole-4-yl) acetate-treated A549 and NCI-H460 cell lines compared to untreated cells (Figure 4). Further, we studied the gene and protein expression of caspase 9 and caspase 3 involved in the intrinsic apoptosis pathway. The gene expression of caspase 9 was upregulated, the protein expression of procaspase 9 was downregulated, and active caspase 9 showed increased expression (Figure 5). Caspase 3 gene expression was found to be increased, the protein expression of procaspase 3 decreased, and active caspase 3 increased in the NSCLC cell lines (Figure 6). These results confirm the involvement of oxidative stress in mitochondrial damage-mediated apoptosis.

### 3.5 Morphological changes during sirtuin 6 inhibition in NSCLC cell lines

**FIGURE 7:**
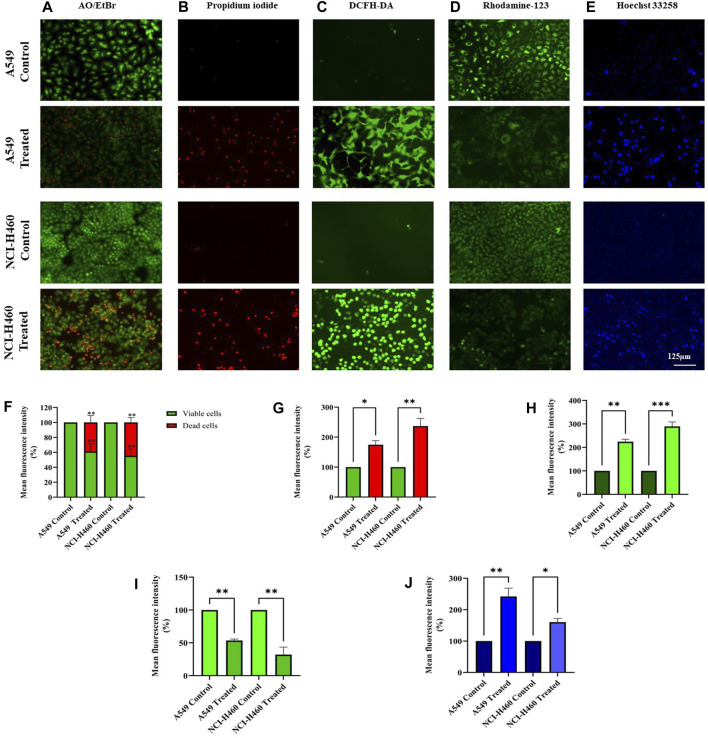
Morphological assessment of NSCLC cell lines A549 and NCI-H460 cells stained by **(A)** AO/EtBr, **(B)** Propidium iodide, **(C)** DCFH-DA, **(D)** Rhodamine-123, and **(E)** Hoechst 33258 were captured using a fluorescent microscope with ×20 magnification (Scale Bar - 125 μm). **(F)** AO/EtBr, **(G)** Propidium iodide, **(H)** DCFH-DA, **(I)** Rhodamine-123, and **(J)** Hoechst 33258 represent the analysis of morphological staining by image **(J)** (Data represent mean values ±SD. *p < 0.05, **p < 0.01 and ***p < 0.001).

We initially performed AO/EtBr staining to examine the apoptosis axis upon SIRT6 inhibition. The green fluorescence represents healthy viable cells with intact membranes, orange fluorescence represents early apoptotic cells, and red fluorescence indicates late apoptosis. Propidium iodide (PI) dye selectively binds to DNA by intercalating between the base pairs. As PI is impermeable to live cells with intact plasma membranes, it can stain dead or damaged cells. Thus, images of live cells appear black without any red fluorescence and images of damaged and dead cells appear red due to the emission of red fluorescence. Mitochondrial membrane potential (ΔΨm) was assessed using rhodamine-123 dye. Rhodamine-123 dye is highly sensitive to changes in mitochondrial membrane potential. Rhodamine-123 staining shows that healthy cells with intact mitochondria have an intense fluorescence emission whereas cells with mitochondrial damage have reduced fluorescence emission. DCFH-DA staining was performed to determine whether the SIRT6 inhibition generates ROS. The DCFH-DA is oxidized to the fluorescent compound called 2′,7′-dichlorofluorescein (DCF). DCF emits a green fluorescence and the intensity of the fluorescence is proportional to the level of ROS present in the cells. The results confirm that the Ethyl 2-[5-(4-chlorophenyl)-2-methyl-1-H-Imidazole-4-yl) acetate-treated cells emitted high-intensity green fluorescence compared to untreated cells. Hoechst dyes are often used to stain the cell nuclei to visualize the nuclear morphology and DNA damage within cells. Hoechst staining emits blue fluorescence when it binds to DNA. The results show highly intense blue fluorescence emission in the Ethyl 2-[5-(4-chlorophenyl)-2-methyl-1-H-Imidazole-4-yl) acetate-treated cells. Overall, the results confirm the impact of SIRT6 inhibition on morphological changes related to ROS generation, mitochondrial damage, nuclear damage, and apoptosis (Figure 7).

### 3.6 Nrf2/Keap1 regulation upon sirtuin 6 inhibition

**FIGURE 8:**
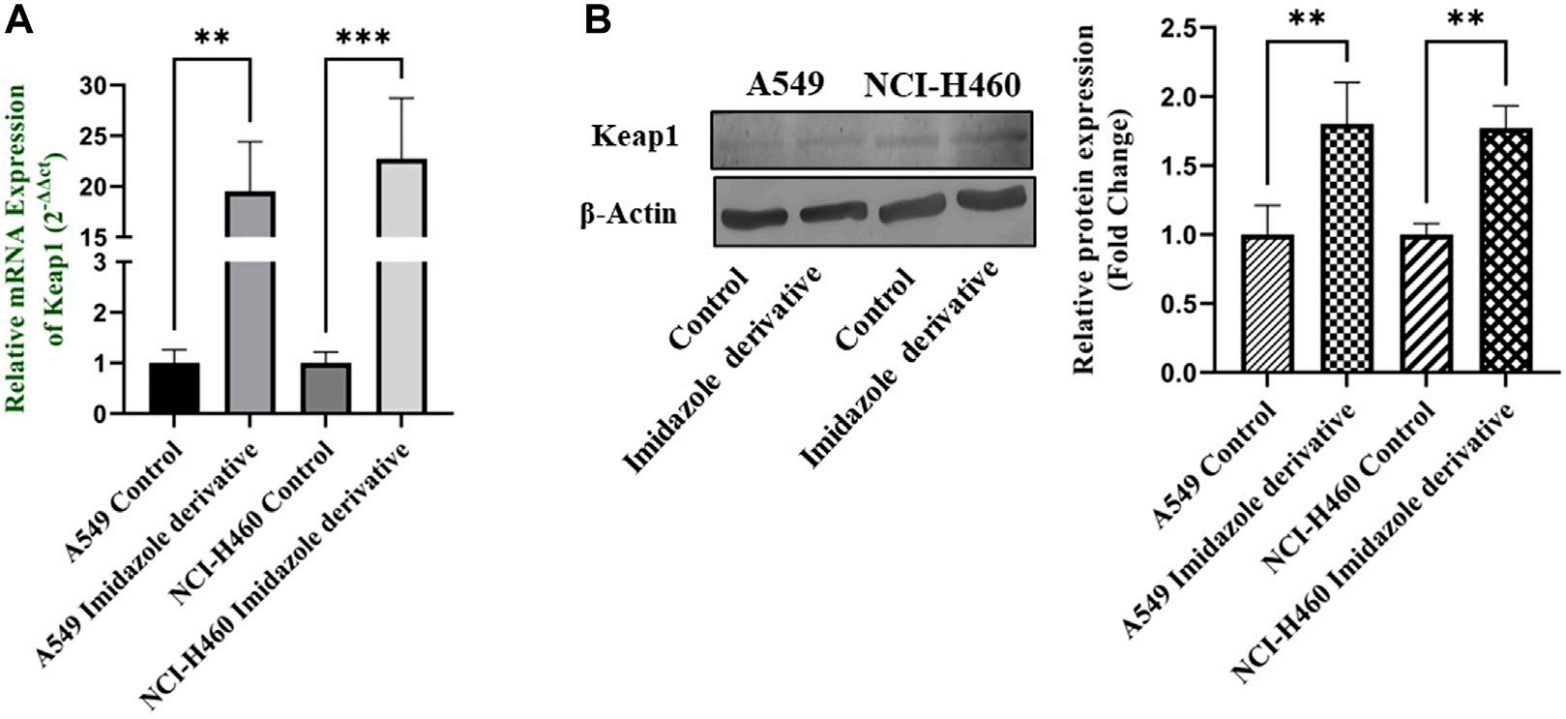
Gene and protein expression of Keap1 in A549 and NCI-H460 NSCLC cell lines: **(A)** Keap1 gene expression and **(B)** Western blot analysis of Keap1. GAPDH for gene expression and β-actin for protein expression were used as an internal control for normalization (Data represent mean values ±SD. **p < 0.01 and ***p < 0.001).

**FIGURE 9:**
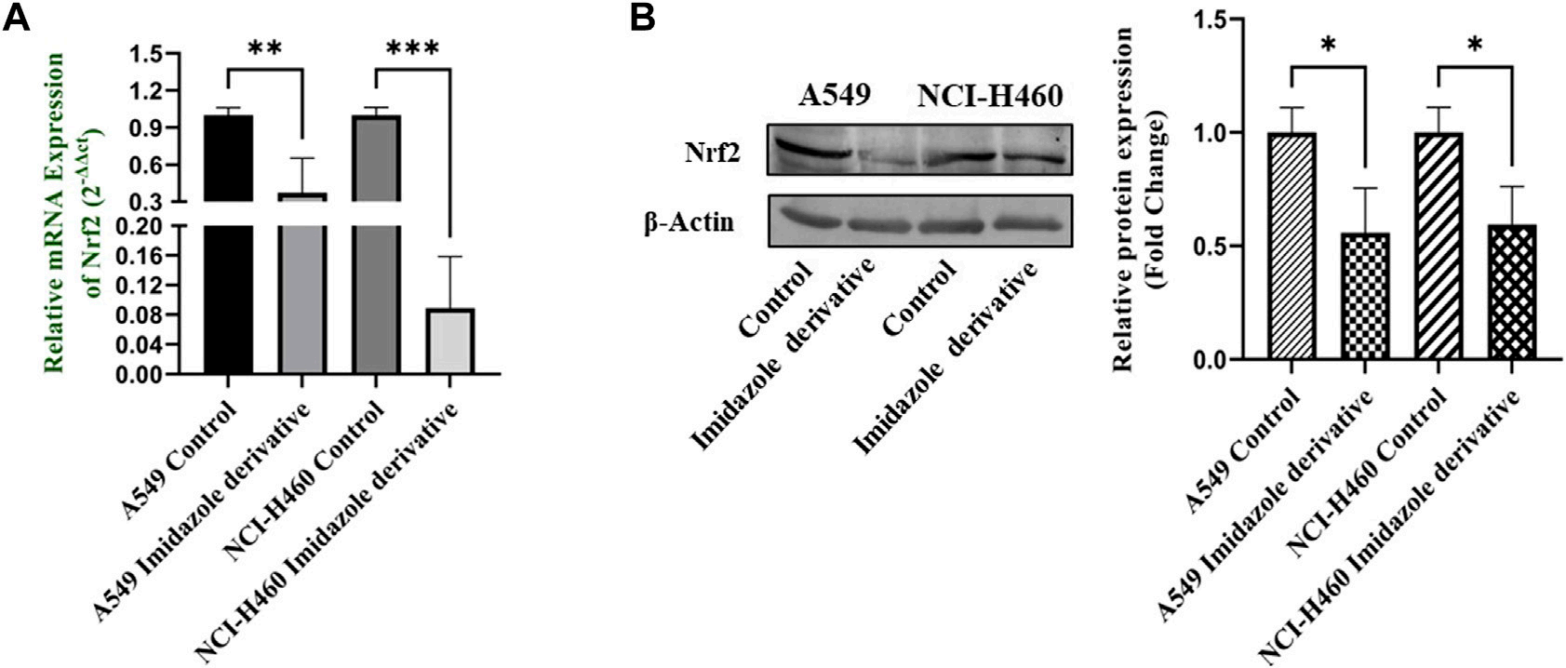
Gene and protein expression of Nrf2 in A549 and NCI-H460 NSCLC cell lines: **(A)** Nrf2 gene expression and **(B)** Western blot analysis of Nrf2. GAPDH for gene expression and β-actin for protein expression were used as an internal control for normalization (Data represent mean values ±SD. *p < 0.05, **p < 0.01 and ***p < 0.001).

Nrf2/Keap1 signaling activates an antioxidant mechanism to prevent oxidative stress, thereby protecting the cells from death. Nrf2 is majorly involved in the activation of these mechanisms whereas Keap1 binds to Nrf2 and causes ubiquitin-mediated proteasomal degradation of Nrf2 in the cytosol. Since SIRT6 is found to be very well involved in the regulation of Nrf2, we studied the expression of Nrf2/Keap1 by inhibiting SIRT6 with Ethyl 2-[5-(4-chlorophenyl)-2-methyl-1-H-Imidazole-4-yl) acetate in NSCLC cell lines. The Keap1 gene and protein expression were found to be upregulated in cells treated with Ethyl 2-[5-(4-chlorophenyl)-2-methyl-1-H-Imidazole-4-yl) acetate in comparison with the control (untreated) cells (Figure 8) whereas Nrf2 gene and protein expression were found to be downregulated by Ethyl 2-[5-(4-chlorophenyl)-2-methyl-1-H-Imidazole-4-yl) acetate treatment in comparison with control (untreated) cells (Figure 9).

## 4 Discussion

Epigenetic dysregulation is a common mechanism in the onset of cancer. Gene regulation is often maintained by the epigenetic modifications associated with DNA methylation and histone methylation/acetylation (Sharma et al., 2009). The role of chromatin-modifying enzymes, particularly sirtuins, has been extensively studied in the initiation and progression of cancer. The overexpression of SIRT6 possesses oncogenic effects and favors the development and progression of tumor cells and thus has become a target for epigenetic therapy. Numerous studies were in progress to identify novel compounds targeting SIRT6 that may provide a new approach to the development of epigenetic therapy. The discovery of imidazole in the 1840s prompted extensive research in drug discovery, focusing on imidazole-based compounds. Imidazole, an important heterocyclic compound, and a core component of FDA-approved drugs, has demonstrated favorable activities in practical applications. It serves as a fundamental building block in numerous natural and synthetic bioactive compounds. The imidazole scaffold offers advantages in binding with various receptors, proteins, and enzymes (Rashid et al., 2023). Imidazole exhibits diverse pharmaceutical activities, including anticancer, antiviral, antibacterial, antifungal, antihistaminic, anti-inflammatory, and other medicinal properties. Notably, imidazole has proven to be an effective anticancer agent. Several imidazole derivatives, such as nilotinib, tipifarnib, zoledronic acid, mercaptopurine, dacarbazine, and temozolomide, are currently employed in treating various cancers. Due to its extensive pharmacological properties, researchers have explored derivatives of imidazole to enhance efficiency and reduce side effects, aiming to overcome the limitations of currently available clinical drugs (Ali et al., 2017).

In the present study, we explored the effect of an imidazole derivative, Ethyl 2-[5-(4-chlorophenyl)-2-methyl-1-H-Imidazole-4-yl) acetate, on SIRT6 inhibition to modulate the Nrf2/Keap1 signaling pathway. This compound possesses various functional groups including ethyl, acetate, chlorophenyl, and methyl, with imidazole serving as the structural backbone. The presence of these functional groups might be a benefit in terms of improved bioavailability and cell membrane permeability, enhanced drug stability and solubility, increased lipophilicity, and metabolic stability in the compound, potentially augmenting the compound’s binding affinity and selectivity, fitting the molecule into the target binding site. First, we found the half maximal inhibitory concentration of Ethyl 2-[5-(4-chlorophenyl)-2-methyl-1-H-Imidazole-4-yl) acetate through an MTT assay. The IC_50_ was found to be 250 µM in A549 and 300 µM in NCI-H460 cell lines. To explore the effect of imidazole derivative on SIRT6 we carried out gene and protein expression studies after treatment with the imidazole derivative and compared it with the control. The gene and protein expression of SIRT6 was found to be downregulated in Ethyl 2-[5-(4-chlorophenyl)-2-methyl-1-H-Imidazole-4-yl) acetate treatment. This shows that the imidazole derivative has an inhibitory effect on SIRT6.

Cancer cells exhibit a higher rate of ROS generation and experience a modified redox environment compared to normal cells (Glasauer and Chandel, 2014). Redox cellular homeostasis is a balanced ratio of the rate and quantity of oxidant generation to the rate of oxidant detoxification. The imbalance between oxidant generation and oxidant detoxification results in oxidative stress (Landriscina et al., 2009). Managing oxidative stress involves maintaining a balance between ROS production and antioxidant defense systems. ROS includes free radicals such as hydroxyl (HO*) and superoxide (O2 *) and non-radical molecules such as hydrogen peroxide (H_2_O_2_) (Perillo et al., 2020). Antioxidants are the molecules that precisely regulate redox balance. These antioxidant molecules exist in enzymatic and non-enzymatic forms. The enzymatic antioxidants act by breaking down and removing free radicals whereas non-enzymatic antioxidants act by interfering with radical chain reactions (Nimse and Pal, 2015). The enzymatic antioxidant defense systems are regulated by superoxide dismutase (SOD), glutathione peroxidase (GPx), and catalase (CAT). Superoxide dismutase (SOD) acts by dismutation of two molecules of O2− into hydrogen peroxide (H_2_O_2_) and molecular oxygen (O2) (Kharrazi et al., 2008). GPx catalyzes the reduction of H_2_O_2_ and lipid hydroperoxides by using reduced GSH, and CAT too involved in the conversion of H_2_O_2_ to water (Aslani and Ghobadi, 2016). Ascorbic acid (vitamin C), α-tocopherol (vitamin E), GSH, and β-carotene are the non-enzymatic anti-oxidant molecules (Kharrazi et al., 2008). GSH is a tripeptide of cysteine, glycine, and glutamate amino acids. It serves as a cofactor for several antioxidant enzymes, including glutathione peroxidase and regeneration of other essential antioxidants such as vitamin C (ascorbic acid) and vitamin E (α-tocopherol) (Aslani and Ghobadi, 2016). Since oxidative stress mechanisms favor cancer cell survival, SIRT6 was reported to be involved in regulating oxidative stress mechanisms. We investigated the impact of SIRT6 inhibition by an imidazole derivative on oxidative stress mechanisms. The biochemical analysis of enzymatic antioxidant molecules such as GPx and CAT, non-enzymatic antioxidant molecule GSH, and the morphological examination of ROS generation by DCFH-DA dye in the imidazole derivative-treated A549 and NCI-H460 cell lines supported the ROS generation and accumulation in NSCLC cell lines. Further, the decreased levels of the essential antioxidant molecules support the decrease in free radical scavenging activity confirmed by the percentage radical scavenging activity assay.

ROS levels below and above the threshold range have the opposite impact on cancer. The ROS levels below the threshold range contribute to the onset, advancement, angiogenesis, and metastasis of tumors by instigating DNA mutations, genomic instability, and abnormal pro-tumorigenic signaling. Various cancer types, such as breast, lung, liver, and others, have shown a correlation with ROS-induced cell proliferation (Glasauer and Chandel, 2014; Navaneetha Krishnan et al., 2019). Conversely, when ROS levels exceed the threshold, they become detrimental to cancer cells, triggering oxidative stress-induced signals that result in cell cycle arrest, senescence, and apoptotic cell death (Chio and Tuveson, 2017). ROS-generating drugs have superior effects against several types of cancer cells through apoptosis induction (Wang et al., 2019). Apoptosis is a programmed cell death critical for maintaining cellular homeostasis. Defects in apoptosis favor cancer cell survival and are of two types: extrinsic and intrinsic apoptosis. The intrinsic apoptosis pathway is activated by genotoxic stress due to unfolded protein response, reactive oxygen species, radiation, and chemical-induced chromosomal abnormalities (Singh and Lim, 2022). Since the ROS levels were accumulated on SIRT6 inhibition by imidazole derivative, we investigated whether our imidazole derivative-based SIRT6 inhibitor was able to induce apoptosis in NSCLC cell lines. Thus, we focused on the molecular expression studies of key regulators that mediate the intrinsic apoptosis pathway such as cytochrome-c, caspases 9, and caspase 3. The release of cytochrome-c and its accumulation in the cytosol initiate the caspase 9 activation. Activated caspase 9, in turn, activates caspase 3, the end effector molecule of the apoptosis cascade, ultimately leading to cell death. The protein expression results confirm that the cytochrome-c was high in the imidazole derivative-treated cells compared to control cells. Further, gene expression studies confirmed the increase in caspase 9 and caspase 3 expression, and the protein expression studies showed a decrease in the expression of pro-caspase 9 and pro-caspase 3 and increased expression of active caspase 9 and active caspase 3. Thus, the results from molecular expression studies of intrinsic pathway regulators confirmed the apoptosis. Further, we performed morphological studies for the assessment of apoptosis in the NSCLC cell lines. AO/EtBr confirms the occurrence of cell death at different stages such as early apoptosis and late apoptosis. The viable cells show uniformly distributed green color with organized chromatin structures. In apoptotic cells, the chromatin is condensed or fragmented, with yellowish-green fluorescence representing early apoptosis and orange nuclear ethidium bromide indicating late apoptosis. Necrotic death is indicated by uneven orange-red fluorescence. A propidium iodide stain is used to assess the live and dead cells. PI is not permeable to viable cells, so the viable cells appear black due to no red fluorescence, whereas dead cells appear red due to PI entry into damaged cells and stain DNA by intercalating between the bases with little or no sequence preference. Hoechst 33258 dye is employed to examine the condensation and fragmentation of nuclei, which are characteristics of apoptosis. The results revealed that the control cells were uniformly stained and had regular, round-shaped nuclei. The treated cells became highly condensed and appear as bright, compacted spots. The nuclei showed signs of fragmentation, with multiple smaller nuclei, also called apoptotic bodies. Mitochondria is a key regulator of apoptosis, in particular the intrinsic apoptosis pathway, which includes a cascade of events such as a change in mitochondrial membrane potential, cytochrome-c release, and activation of caspase molecules (Zhao et al., 2016). Rhodamine-123 green-fluorescent dye measures changes in mitochondrial membrane potential. The results show that the control cells with intact mitochondria accumulated rhodamine-123, leading to strong fluorescence whereas the treated cells had reduced rhodamine-123 uptake and lower fluorescence, confirming the decrease in mitochondrial membrane potential. The morphological studies confirm the imidazole derivative’s role in the decrease in mitochondrial membrane potential, ROS generation and accumulation, nuclear damage, and apoptosis cell death. Altogether, the results from molecular expression studies of intrinsic pathway regulators and the morphological studies confirm the significant role of the imidazole derivative in apoptosis-induced cell death of A549 and NCI-H460 cell lines.

Nrf2, a crucial transcription factor that regulates cytoprotective responses against xenobiotic/electrophilic and oxidative stress, plays a significant role in cancer development, progression, and resistance (Panieri and Saso, 2019). Nrf2 exerts its influence through the AREs found in the 5′-upstream regulatory regions of most cytoprotective genes, which allows it to modulate oxidative stress (Ulasov et al., 2022). The regulation of Nrf2 is controlled by its suppressor protein, Keap1, acting as an on/off switch in response to redox stimuli. Under normal redox conditions, Keap1 binds to and retains Nrf2 in the cytosol. Keap1 also functions as a substrate adapter for the Cullin 3 (CUL3)-containing E3-ligase complex, leading to the ubiquitination of Nrf2 and its subsequent degradation by the 26S proteasome (He et al., 2023). However, during oxidative stress, specific cysteine residues in Keap1 act as sensors and undergo conformational changes that prevent Keap1 from binding to Nrf2. Consequently, Nrf2 translocates to the nucleus, where it partners with small Maf or other nuclear proteins to bind to ARE, thereby activating genes involved in the antioxidant mechanism (Dinkova-Kostova and Copple, 2023). The accumulation of Nrf2 in cancer cells and its cytoprotective role in cancer can be attributed to several factors. Firstly, somatic mutations in Nrf2, Keap1, or CUL3 have been reported. Secondly, Keap1 downregulation can occur through epigenetic mechanisms. Thirdly, the interaction of p62/Sqstm1 and p21 with Keap1 can influence Nrf2 levels. Finally, cometabolites can modify Keap1 cysteine residues (Taguchi and Yamamoto, 2017). Since Nrf2/Keap1 signaling is reported to be a major regulator of cellular homeostasis, SIRT6 is very well involved in the regulation of Nrf2 by inhibiting Keap1. We investigated the impact of SIRT6 inhibition on Nrf2/Keap1 signaling. Our data shows that Nrf2 was downregulated, whereas Keap1 was upregulated at gene and protein expression levels. The gene and protein expression data of Nrf2 and Keap1, the critical components of Nrf2/Keap1 signaling, confirm that the imidazole derivative affects Nrf2/Keap1 signaling directed through SIRT6 inhibition.

## 5 Conclusion

In conclusion, the overall findings obtained in the study conclude that our novel compound Ethyl 2-[5-(4-chlorophenyl)-2-methyl-1-H-Imidazole-4-yl) acetate has potential anticancer activity, mainly by targeting SIRT6, which interrupts the Nrf2/Keap1 signaling pathway, causing an accumulation of ROS, and resulting in oxidative stress. Moreover, oxidative stress-induced mitochondrial damage triggers the activation of the intrinsic apoptosis pathway in A549 and NCI-H460 cell lines. This study confirms the mechanism of action of Ethyl 2-[5-(4-chlorophenyl)-2-methyl-1-H-Imidazole-4-yl) acetate in inducing apoptosis. Hence, targeting SIRT6 might be a new promising epigenetic therapeutic approach to evade Nrf2/Keap1-powered cellular rescue pathways in lung cancer.

## Data Availability

The original contributions presented in the study are included in the article/Supplementary Material, further inquiries can be directed to the corresponding authors.

## Abbreviations

AO, Acridine orange; ARE, Antioxidant Responsive Element; A549, Adenocarcinoma; CAT, Catalase; cyt-c, cytochrome-c; DCFH-DA, 2 2′,7′-dichlorofluorescin; DPPH, 2,2-diphenylpicrylhydrazyl; DTNB, 5,5′-dithiobis-(2-nitrobenzoic acid) (Ellman’s reagent); EtBr, Ethidium Bromide; GSH, Glutathione; GPx, Glutathione peroxidase; HDAC, Histone deacetylase; HDACi, Histone deacetylase inhibitor; Keap1, Kelch-like ECH-associated protein 1; NSCLC, Non-small cell lung cancer; NCI-H460, Large cell carcinoma; Nrf2, Nuclear factor erythroid 2-related factor 2; ROS, Reactive oxygen species; SIRT6, Sirtuin 6; % RSA; % Radical Scavenging Activity; PI, Propidium iodide.
